# Development of a standardised instrument to assess ageing-related health outcomes in a comparable fashion in European ageing populations, the CHANCES Health Module

**DOI:** 10.1186/1753-6561-7-S4-S7

**Published:** 2013-08-23

**Authors:** Martin Bobak, Simone Croezen

**Affiliations:** 1 Department of Epidemiology and Public Health, University College London (UCL), London, UK; 2 Department of Public Health, Erasmus MC, Rotterdam, the Netherlands

## 

The FP7 Consortium on Health and Ageing: Network of Cohorts in Europe and the United States (CHANCES) is a collaboration project of 14 large cohort studies in Europe and North America. The main aim is to investigate the rates, determinants and implications of cardiovascular diseases, diabetes, cancer, disability, fractures, cognitive functions and mortality in older persons. An important part of the project is the development of a health module to measure the most important age-related health outcomes.

The rationale for developing a short health module is that there is currently a wide range of instruments to measure health in older people. Health surveys have used different measures with varying degrees of validation, resulting in data that are often not comparable between studies, across populations and over time. The aim of the CHANCES module was to review the existing instruments measuring health and health-related variables and to develop a single consolidated battery of measures. This new health module should be self-standing and reasonably short, so that it can be added to existing studies not originally focusing on ageing.

The selection of health domains was based on systematic reviews and analyses of empirical data. The module includes a mixture of subjective and objective measurements of the following domains: general health status, cognitive functions, physical functions, disability, depressive symptoms, physical activity, anthropometry, weight loss, eyesight, hearing, oral health, sleep, quality of life and a blood sample. The objective measurements include cognitive functions (word recall, verbal abilities, executive function) and physical function tests (grip strength, chair rise, walk speed). The time requirement for the full module is under one hour; however, it has a core and an extended component for additional flexibility in terms of scope and timing. The module is currently piloted in 3 geographically diverse populations (Northern Ireland, Greece and Poland). The pilot data will be analysed to assess the timing, data missing and feasibility and examine the agreement between the new and existing instruments in different dimensions of health outcomes. The pilot data analyses may lead to modifications of the final version of the instrument.

The new instrument will provide standardised and comparable information on prevalence of health in elderly populations in Europe. Existing and new studies, not primarily focused on ageing, can use the module to extend the scope to the study of older persons. If the module is successful and is adopted by the research community, the CHANCES project will make an important contribution to comparable assessment of ageing-related health outcomes between populations and over time.

